# Near-surface real-time seismic imaging using parsimonious interferometry

**DOI:** 10.1038/s41598-021-86531-5

**Published:** 2021-03-30

**Authors:** Sherif M. Hanafy, Hussein Hoteit, Jing Li, Gerard T. Schuster

**Affiliations:** 1 Department of Geosciences, King Fahd University of Petroleum and Minerals, CPG, Dhahran, 34464 Saudi Arabia; 2 Division of Physical Science and Engineering, King Abdullah University of Science and Technology, Thuwal, 23955-6900 Saudi Arabia; 3 College of Geo-Exploration Science and Technology, Jilin University, Changchun, China

**Keywords:** Geophysics, Hydrogeology

## Abstract

Results are presented for real-time seismic imaging of subsurface fluid flow by parsimonious refraction and surface-wave interferometry. Each subsurface velocity image inverted from time-lapse seismic data only requires several minutes of recording time, which is less than the time-scale of the fluid-induced changes in the rock properties. In this sense this is real-time imaging. The images are P-velocity tomograms inverted from the first-arrival times and the S-velocity tomograms inverted from dispersion curves. Compared to conventional seismic imaging, parsimonious interferometry reduces the recording time and increases the temporal resolution of time-lapse seismic images by more than an order-of-magnitude. In our seismic experiment, we recorded 90 sparse data sets over 4.5 h while injecting 12-tons of water into a sand dune. Results show that the percolation of water is mostly along layered boundaries down to a depth of a few meters, which is consistent with our 3D computational fluid flow simulations and laboratory experiments. The significance of parsimonious interferometry is that it provides more than an order-of-magnitude increase of temporal resolution in time-lapse seismic imaging. We believe that real-time seismic imaging will have important applications for non-destructive characterization in environmental, biomedical, and subsurface imaging.

## Introduction

Many systems of interest are dynamically changing over short periods of time. Some examples include engineering materials under dynamic loading, leaky dams with hidden fractures^1^, living organisms with temporal variations in blood flow and temperature, eruptions of lava along fissures or volcanic vents^2^, geysers that vent steam and hot water every few minutes or hours^3,4^, carbon capture and sequestration type projects at least in the first phase of the operations or the recent discovery of ice plumes erupting from Europa^5^. An engineering example is that of decaying dams, where hidden water leakage into aging dams can lead to disastrous dam failure^1^. In general, the scale of temporal variations can be proportional to the size of the object, which can lead to significant property changes from a few minutes to months for fluid flowing through the porous medium.

The problem with seismic imaging for probing rapidly changing properties of the Earth is that each seismic experiment can take from hours to days to complete. Larger time-lapse surveys provide greater subsurface coverage but at the cost of a dramatic decrease in temporal resolution of time-lapse images. For a typical engineering survey, it typically takes several hours or more to carry out an experiment with several hundred recording stations and a source excited at each station. Here, we assume that the seismic images are extracted from raw seismic data by a tomographic technique such as traveltime tomography or surface-wave inversion^6^. This means that the typical temporal resolution of time-lapse imaging is limited to an hour or more. Such restrictions can be unacceptable, for example, if hidden water leakage in an aging dam is not detected in time to prevent a catastrophic dam failure^1,7^. Another need for rapid time-lapse monitoring is for predicting imminent mine collapses^8^ and for the location of trapped miners^9^.

To rapidly generate shot gathers in less than one hour, Hanafy and Schuster^10^ introduced parsimonious interferometry (PI) for refractions. The PI method creates many virtual refraction shot gathers from just a few infill shot gathers and two reciprocal shot gathers recorded at both ends of the recording line. For refractions, the assumptions are that the first arrivals mainly consist of head waves and direct waves. Refraction traveltimes from two reciprocal shot gathers (excited by sources at the two ends of the recording line) and a few infill shot gathers are picked, and parsimonious interferometry can be used to compute O(N^2^) refraction traveltimes generated by *N* virtual sources. Here, *N* is the number of geophones in the 2D survey. The resulting refraction traveltimes can then be inverted by refraction tomography to produce tomographic snap-shots of the subsurface velocity model every few minutes. This enormous increase in the number of traveltime picks and associated rays, compared to the many fewer traveltimes from the two reciprocal and several infill shot gathers, provides for increased model resolution and a rapid delineation of the fluid-flow properties^10^ in the subsurface. Parsimonious interferometry can also be used for rapidly reconstructing the S-velocity model from recorded surface waves, as demonstrated by^11^.

The problem with PI is that it is not yet validated with controlled time-lapse experiments where the subsurface properties vary significantly over time. We now validate the efficacy of PI with a controlled-source experiment where 12 tons of water are injected into the subsurface over a period of 4.5 h. Seismic traces are recorded for a few shots every two minutes or less, where each shot is recorded by 72 geophones spaced at 0.5 m intervals. Then, PI is used to transform these few shot records into 72 virtual shot records, each with 72 virtual traces. Each virtual shot record contains 72 first-arrival traveltimes as if they were generated by an actual source. These data are inverted by refraction (first-arrival) traveltime tomography to give time-lapse images of the subsurface to a temporal resolution of 2 min. As we will show, the time-lapse tomograms are in qualitative agreement with 3D fluid simulations and laboratory experiments. We also apply surface-wave parsimonious interferometry^11^ to the Rayleigh waves and invert for the S-wave velocity tomogram. Both the Vp and Vs tomograms can then be used to compute the time-varying Vp/Vs tomograms, which indicates the degree of temporal viriations of water saturation in the subsurface.

## Method: Refraction parsimonious interferometry

**Figure 1:**
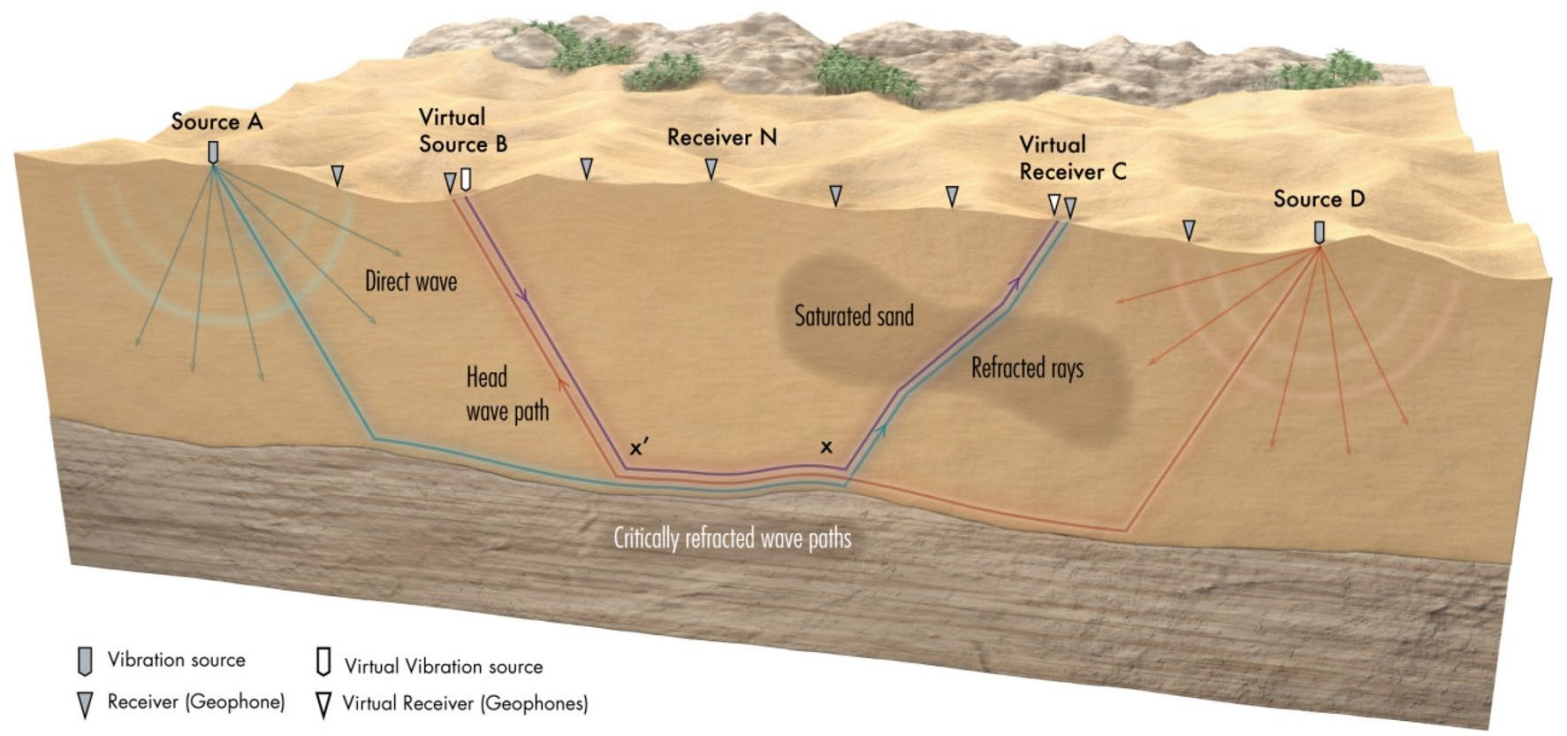
Refraction ray diagrams for the parsimonious refraction interferometry method and a 2-layer model, where the lower layer has a faster seismic velocity than the upper layer.

For a 2D seismic experiment, assume a point source at each end of a straight recording line and the irregularly layered medium shown in Fig. 1, where head waves propagate along the boundary between the first and the second layers. For convenience, we assume a two-layer model but the method is valid for a many-layered model with lateral velocity variations as long as the velocity mostly increases with depth. Here, there are *N* geophones placed at the recording surface between the two reciprocal sources located at ***A*** and ***D***. The head-wave traveltime from the source at ***A*** to a receiver at ***C*** is defined as:

1 $${\tau }_{AC}={\tau }_{A{x^\prime}}+{\tau }_{{x^\prime}x}+{\tau }_{xC},$$

and the opposite traveltime from D to B is given by

2 $${\tau }_{DB}={\tau }_{Dx}+{\tau }_{{x^\prime}x}+{\tau }_{{x^\prime}B},$$

where ${\tau }_{xy}$ is the first-arrival traveltime from *x* to *y* along the refraction ray. To create virtual sources and receivers within the array in Fig. 1, we assume that the geophones located at positions ***C*** and ***B*** are separated at a post-critical distance and both of them record head waves from the same interface. Adding Eqs. (1) to (2) and subtracting the reciprocal traveltime (${\tau }_{AD}$), gives the interferometric stationary traveltime for a virtual wave to propagate from a virtual source^12^ located at ***B*** to a receiver located at ***C***:

3 $${\tau }_{CB}={\tau }_{AC}+{\tau }_{DB}-{\tau }_{AD}=\left[{\tau }_{A{x^\prime}}+{\tau }_{{x^\prime}x}+{\tau }_{xC}\right]+\left[{\tau }_{Dx}+{\tau }_{{x}^{\mathrm{^{\prime}}}x}+{\tau }_{{x^\prime}B}\right]-\left[{\tau }_{A{x^\prime}}+{\tau }_{{x^\prime}x}+{\tau }_{xD}\right]={\tau }_{{x^\prime}B}+{\tau }_{{x^\prime}x}+{\tau }_{xC}.$$

The above formula is then used to compute the O(*N*^2^) refraction traveltimes for a virtual source at any of the *N* geophones using the *2N* traveltimes generated by the two reciprocal shot gathers. This is true only if the offset between the two receivers ***B*** and ***C*** is at a post-critical distance. However, if the offset between the two receivers ***B*** and ***C*** is smaller than the post-critical distance, then Eq. (3) should not be used and we fill-in the missing traveltimes by interpolating the direct waves picked from the infill shot gathers^10^. Additional details and numerical simulations of the refraction PI method are given in^10^.

### Parsimonious surface-wave interferometry method

Similar to virtual refraction arrivals generated by the PI method, virtual shot gathers containing surface waves can also be generated at every geophone in the recording survey^11^. Details for generating the virtual surface waves are given in^11^, the general theory is in^12^ and the Supplemental Information section.

## Results

**Figure 2:**
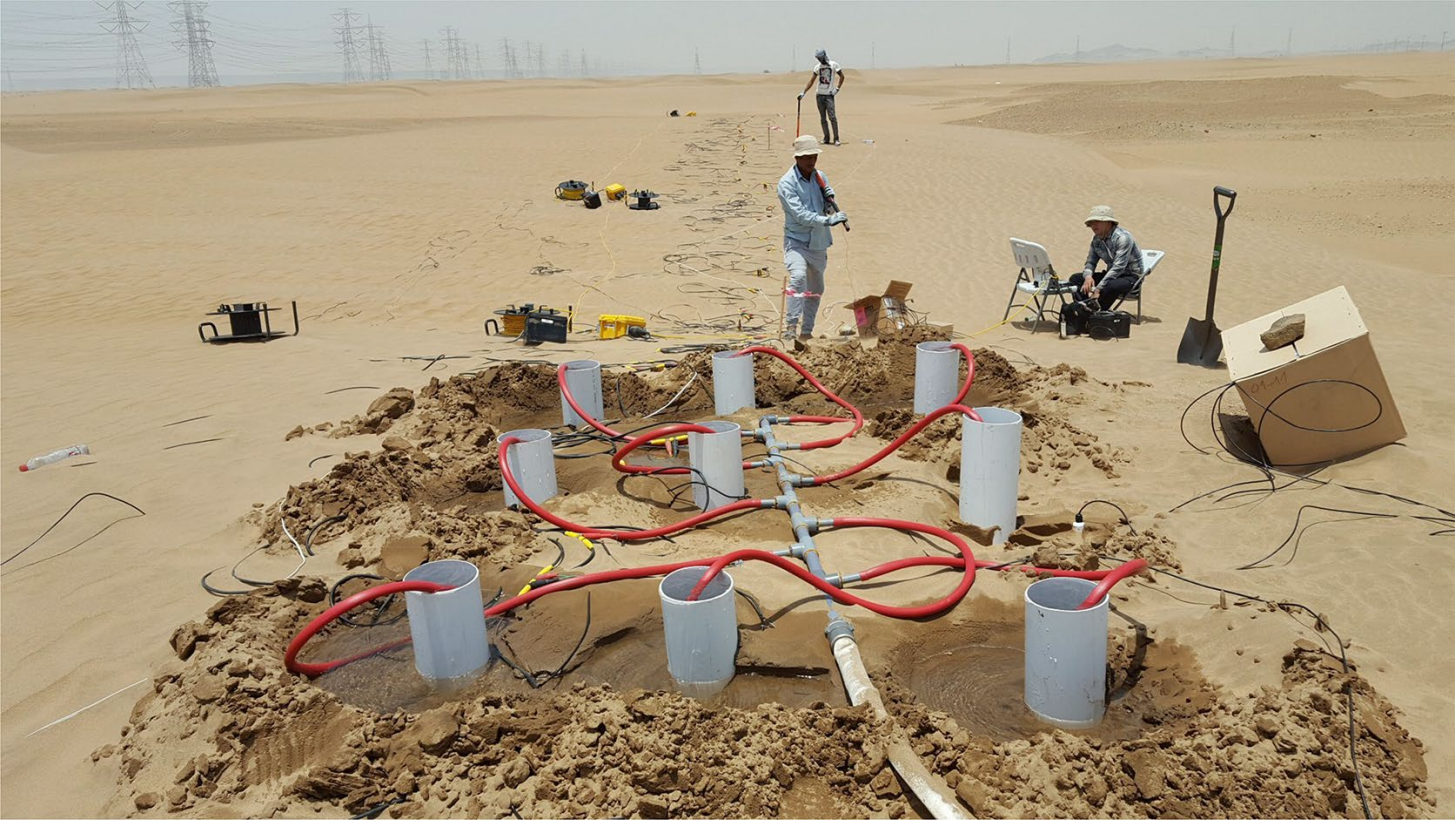
Photograph of the field experiment where the seismic data are recorded over the sand dune during the injection of 12-tons of water.

The time-lapse parsimonious interferometry method is now tested on seismic data recorded over a sand dune (Fig. 2) near the KAUST campus where 12 tons of water were injected over a 4.5-h period. Here we invert for the P-wave and S-wave velocity models from each of the time-lapse data sets to monitor the changes in the subsurface velocity.

We selected a dry sand dune that could be considered as a vadose zone, which is an important challenge for hydrological characterization^13,14^. Hydrological characterization can be accomplished by using geophysical techniques to image the subsurface hydrogeological structures at appropriate scales^14^. In addition, time-lapse geophysical methods have proven useful for monitoring flow and water content dynamics^15^.

### Field data test

Prior to the water injection, we recorded a reference data set with 72 shot gathers recorded by 72 receivers per shot gather with a 0.5 m geophone spacing. The reference data are inverted, and the resulting P-velocity tomogram is used as ground truth for the parsimonious interferometry (PI) method (see Supplemental Information section). Each shot is excited using a 10-kg sledgehammer hitting a metallic plate near every 40-Hz geophone that records the vertical particle velocity.

We started by recording two conventional seismic data sets, where all 72 shot gathers are recorded by exciting two shots at each geophone location and stacking together the corresponding two-shot gathers. The first-arrival traveltimes are picked and inverted to estimate the background velocity model prior to water injection. Each conventional data set required about 2 h to be completed; the conventional data will also be referred to as the background data. Then, 12 tons of water were injected into the subsurface over a period of 4.5 h, while we recorded 90 different PI data sets. Each PI data set consisted of 6 shot gathers with 72 traces per shot gather, and the shots are located at receivers 1, 15, 29, 43, 57, and 72. The first-arrival traveltimes from the 6 shot-gathers are picked and used to generate the virtual traveltimes for 66 virtual shot gathers using Eq. (3). Each data set consists of (66 virtual + 6 active) × 72 = 5184 traveltimes and required an average of about 2 min in recording and deployment time. This is almost 2% of the time required to record a conventional data set consisting of 72 shot gathers (i.e., 72 × 72 = 5184 traveltimes) with a shot at each geophone location. The virtual traveltimes prior to water injection are mostly within about 3 ms of those picked from the background traveltimes.

### Time-lapse experiment and velocity tomograms

The PI method is used to calculate the virtual refraction traveltimes and fundamental-mode Rayleigh waves of 66 (= 72–6) virtual shot gathers for all 90 data-sets recorded in the field. The goal is to compute the real-time P-velocity and S-velocity tomograms from the virtual shot gathers in order to track the flow of water beneath the sand dune. Changes in the P-wave and S-wave velocity tomograms should reflect the flow of water and its injection on the surface.

**Figure 3:**
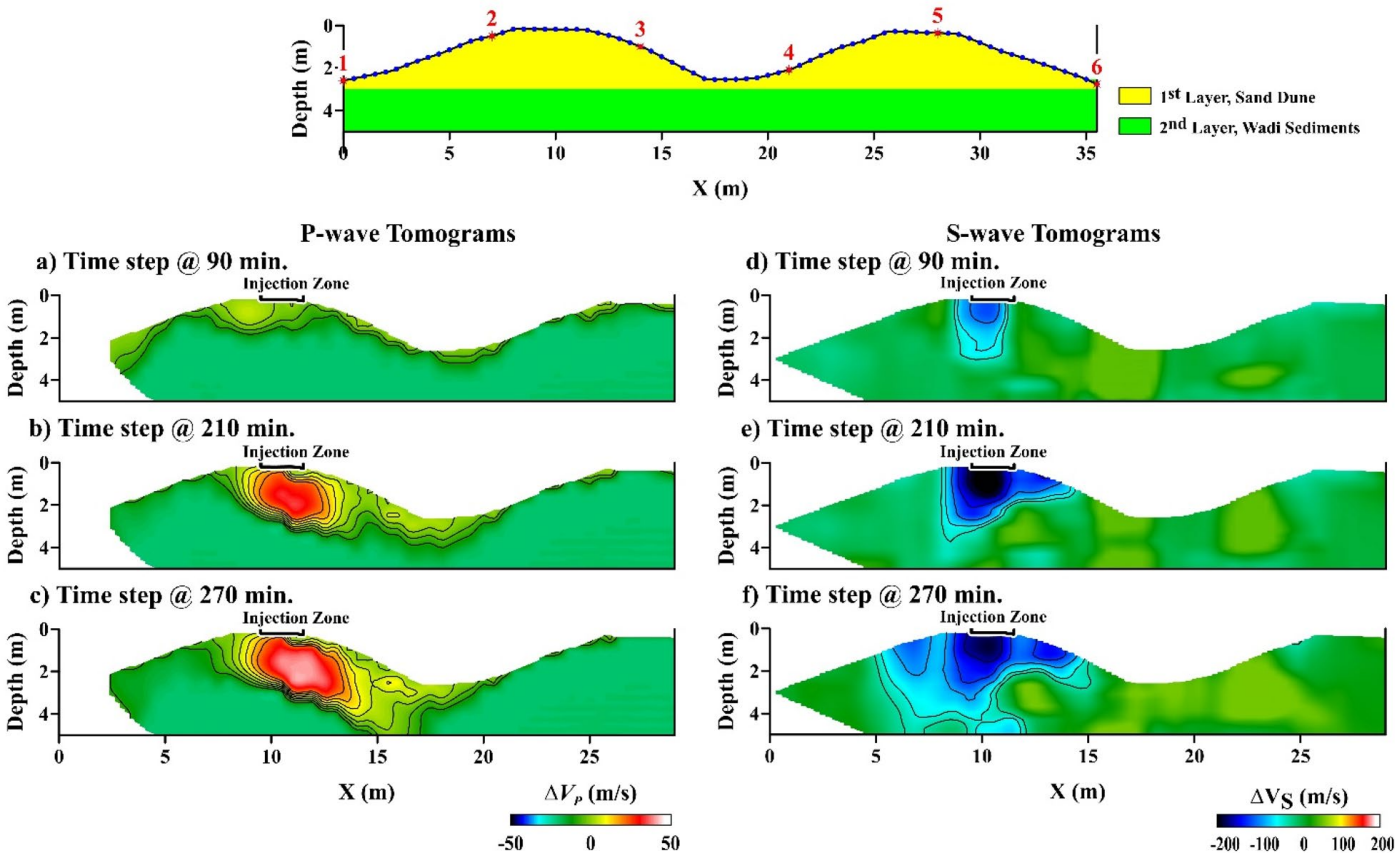
Time-lapse difference tomograms computed by parsimonious interferometry. The top diagram depicts the configuration of the six-sources (red asterisks) and 72-receivers (blue dots) located at the top of the sand dune (yellow layer). Figures (**a**–**f**) are the velocity changes, relative to the background tomograms computed prior to water injection, in the (left column) P-velocity and (right column) S-velocity tomograms for different recording times. Surfer 11 (https://www.goldensoftware.com/products/surfer) is used to plot this Figure.

**Figure 4:**
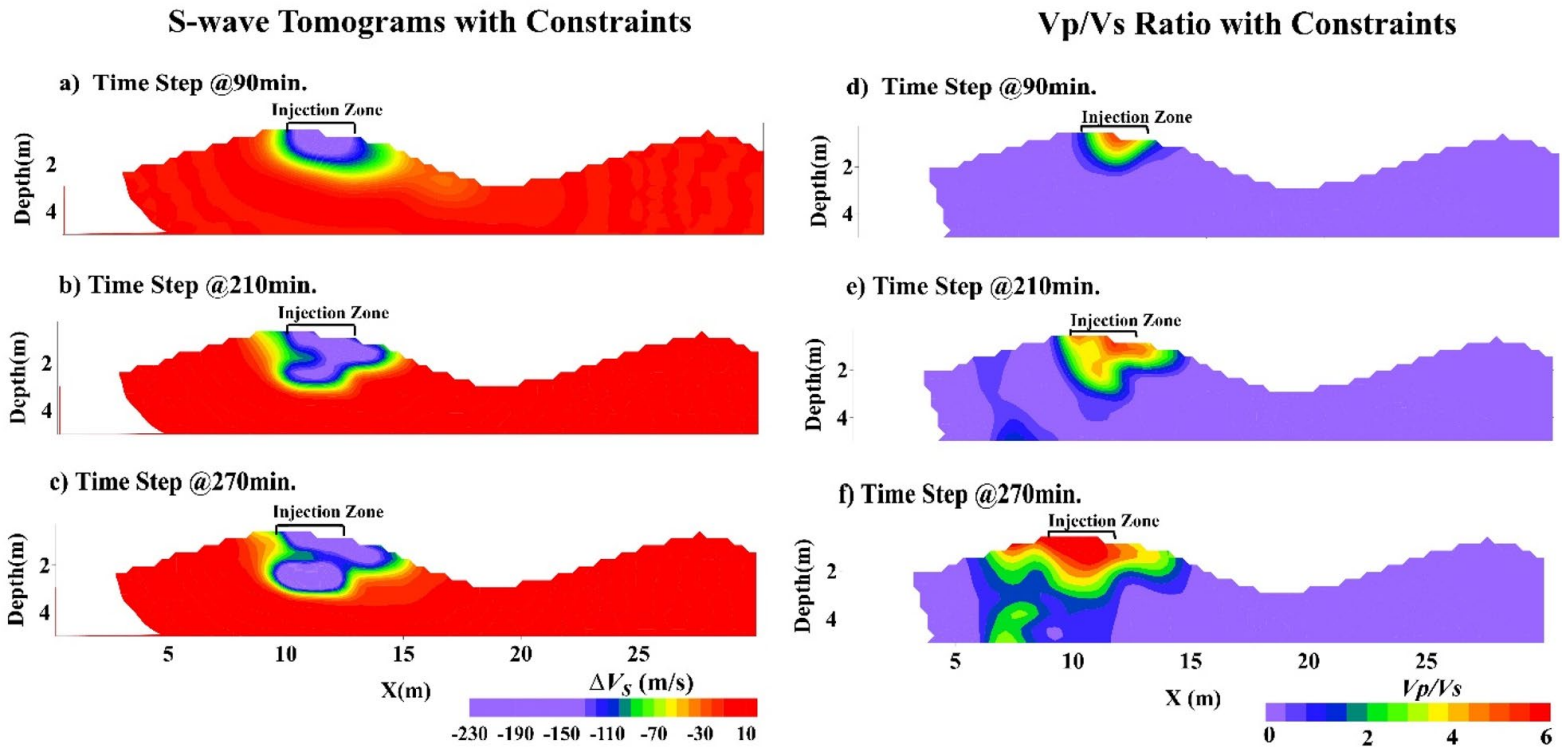
S-velocity tomograms in the left column have been computed using the gradient regularization constraint described in the text and the right column consists of the Vp/Vs ratio tomograms using the Vp and Vs tomograms in the left columns of Figs. 2 and 3, respectively. Surfer 11 (https://www.goldensoftware.com/products/surfer) is used to plot this Figure.

First, the virtual shot gathers are computed from the 6 actual shot gathers (Fig. 3 top) recorded every few minutes. These virtual shot gathers are used to generate 66 virtual shot gathers of traveltimes and 66 surface-wave dispersion curves. The virtual traveltimes and fundamental-mode dispersion curves are inverted to calculate the P- and S-velocity tomograms at about two-minute intervals. These tomograms are subtracted from the background velocity tomogram to get the velocity variation tomograms shown in Fig. 3. To compute the Vp/Vs ratio tomograms in Fig. 4d–f, we used a constrained inversion for the S-velocity tomograms^16^ (see Supplemental Information material and Fig. 4a–c).

The P-velocity tomograms in the left column of Fig. 3 show an initial increase of 10 m/s in the first 90 min, which increased to about 40 m/s after 210 min from the initial water injection. This represents an average of 11–14% increase in the P-velocity values due to water injection. These changes take place within about the first meter or so of the surface, suggesting that the water has not penetrated below this depth. Temperature gauges inserted at holes surrounding the injection site only showed temperature changes no deeper than 1 m for the first 90 min. In contrast, the S-velocity variations decrease by 200 m/s by 210 min from the initial water injection, which is about 30–35% decrease in the S-velocity values. The increase in P-velocity suggests an increase in water saturation as demonstrated by seismic experiments in sand^17^. In addition, the large decrease in S-velocity is consistent with the laboratory results in^18,19^, which measured a 100–150 m/s decrease in S-wave velocity when water was injected into dry sand. In our case, the large decrease in the S-velocity values could, also, be due to soil liquefaction as a result of water injection. Experimental results suggest that the S-wave velocity in sandstones is inversely correlated with water content where higher water content leads to lower S-velocities^20^. In contrast, P-wave velocities typically increase with higher water saturations in sandstones^21^.

The interpretation of the tomograms in Figs. 3 and 4 suggests that the water is mostly flowing downward and downhill to the right of the injection zone, a hypothesis that is examined by our modeling simulations in the next section.

The right column of images in Fig. 4 depicts the Vp/Vs tomograms, which are computed from the Vp tomograms in Fig. 3 and the constrained^16^ Vs tomograms in Fig. 4a–c (Supplemental Information material). The temporal increase in Vp/Vs is in agreement with the general increase theoretically predicted in^22^: *‘…the Poisson ratio and Vp/Vs are always higher for the fluid saturated porous solid than for its dry counterpart’*. The initial Vp/Vs ratio of the near-surface sand (with porosity values of 0.42–0.45) in our experiment is estimated to be 0.12–0.15 before water injection, and after water injection, the Vp/Vs ratio in our Fig. 4f tomogram increases with saturation to a maximum between 5.0 and 6.5. Even though we cannot say if the sand was fully saturated, our results are in general agreement with the effective medium predictions^22^ of Vp/Vs (see Fig. 2d,h in^22^) for a fluid-saturated rock.

## 3D fluid-flow simulations

Figures 3 and 4 show that the tomograms after 210 min of water injection clearly indicate a mostly right-going infiltration of water towards the bottom of the dune. To further investigate this observation, we used fluid-flow simulations to model the two-phase water–air flow in the dune. The simulator is based on the finite-volume method that solves the governing equations given by Darcy’s law and the mass conservation equations for water and air^23,24^, where the flow mechanisms are related to gravity and capillary forces. We collected sand samples to determine key input parameters for the simulations, which include porosity, permeability, sand/air capillarity, sand distribution, and the 3D structure of the dune.

**Figure 5:**
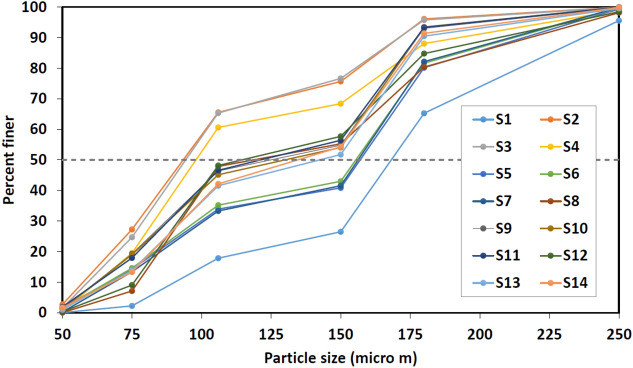
Particle-size distribution for 14 sand samples, labeled as S1, S2, …, S14.

The 3D structure was determined from the surface topography of the dune. The simulation model occupies the volume 18 m × 18 m × 4 m and is described by grids^25^ of size 100 × 100 × 60 in the x- (along the receiver line), y-, and z-directions. The model consists of two formation types: sand dune, and basement, as shown in Fig. 2. The sand dune is assumed to be composed of alternating layers of two different sand types. To characterize the sand, we conducted a particle-size analysis for 14 samples, as shown in Fig. 5. The particle-size range is within 50–250 μm for all samples but exhibits different distributions. The particle-size medians across the samples, appearing at the 50th percentile in Fig. 5, varies between 90 and 160 μm. This distribution reveals at least two types of sands which, can be classified^26^ as very fine sand (62–125 μm) and fine sand (125–250 μm).

### Simulation of the field experiment

The uniformity coefficient, defined by

4 $${U}_{c}=\frac{{d}_{60}}{{d}_{10}},$$

where *d*_*60*_ represents the grain size at which 60% of the sand is finer, varies within 1.8 to 2.5 for all samples. This range of *U*_*c*_ is well below 4, which indicates a uniform particle distribution within individual samples^17^. The porosity measured for all samples was within 0.42 and 0.45, and the permeability was around 24 Darcy, corresponding to a hydraulic conductivity of about 20.5 m/day. The capillary-rise values measured for the fine and very fine samples were, respectively, 34 cm and 64 cm, which indicates a significant capillary contrast between the two samples.

The sand distribution configuration within the domain, reflecting the different sand types, was found to be crucial to capture the observed flow behavior. The structure of sediments in a dune is complex and typically composed of layers and boundary surfaces, where variations in wind deposition may result in a layered grain-size distribution^27–29^. To investigate the impact of the vertical variations in sand grains, we consider a zebra-pattern distribution^30^, where our classified sands, fine and very fine, are alternated in layers.

**Figure 6:**
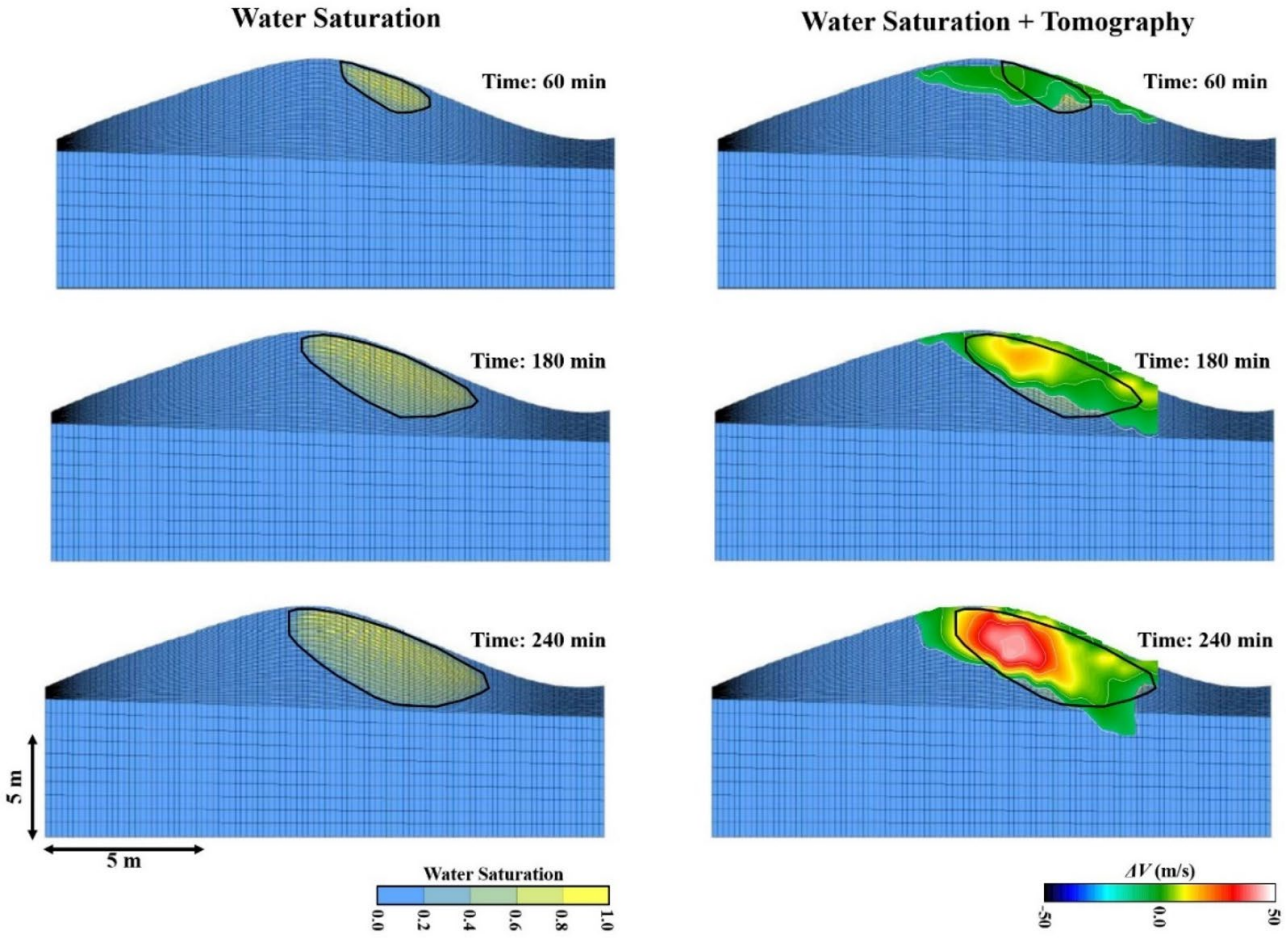
Fluid-flow simulation results showing (left) water saturation and (right) water-saturation + tomogram maps at different times. The images on the right are the P-velocity tomogram images overlain on the black boundary lines of the water-saturation images in the left column. Surfer 11 (https://www.goldensoftware.com/products/surfer) is used to plot this Figure.

Simulation results showing the water saturation maps in a cross-section at different times are given in the left column of images in Fig. 6. The predicted water-infiltration drift to the right is consistent with the tomographic images in the right column. The predominate flow mechanisms are related to the contrast in capillary pressures between the two sand types; sand with finer gains has higher specific retention and capillary force, which tends to suction water laterally following the layer profile, and inclination as a result of gravity. This lateral flow phenomenon in sand dunes has also been observed by^31,32^. The tomographic images in Figs. 3 and 4 are qualitatively consistent with the fluid flow simulations in Fig. 6, which validates the feasibility of our fluid-flow model.

## Conclusions

We demonstrated that parsimonious refraction and surface wave interferometry can be used to estimate the time-varying distributions of the P-wave velocity and S-velocity models. The field data results show that six shot-gathers can be recorded in a few minutes or less, and then be used to generate the first-arrival traveltimes and surface waves from 72 shot gathers. These data are then inverted to give snapshots of the P-wave and S-wave velocity variations at two-minute intervals, which can be considered as real-time imaging compared to the longer time scales of the fluid flow.

**Figure 7:**
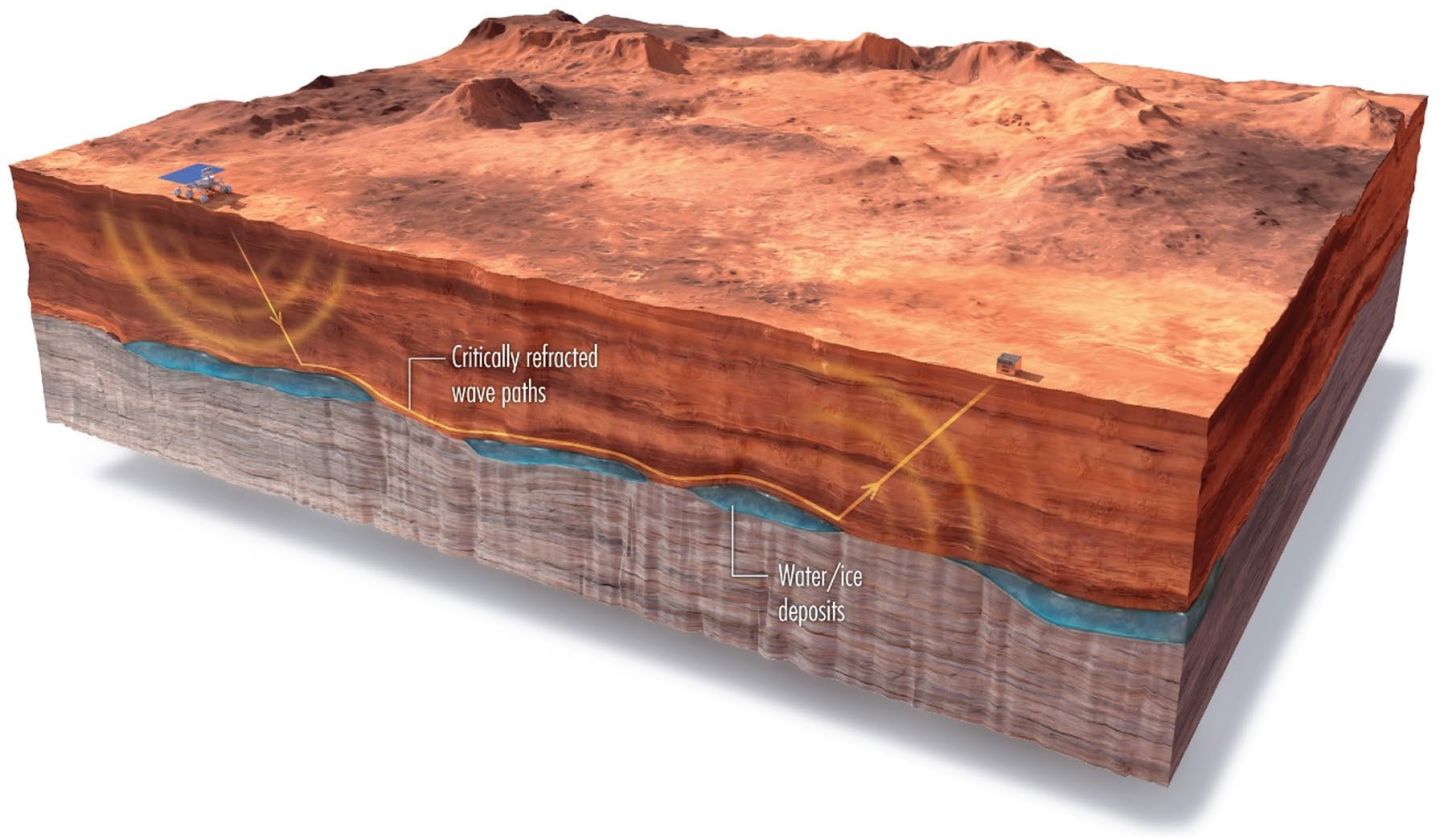
Martian PI experiment. Either geophones or a line of fiber optic sensors^26^ are planted along a survey line on the surface of Mars or the Moon. A land rover or a person strikes the ground at a sparse number of source locations to generate seismic waves that are recorded. The resulting refractions and surface waves are recorded similar to those of the conventional PI survey illustrated in Fig. 1. For a fiber optic recording line, the inline horizontal-component of Rayleigh waves can be recorded by a fast PI survey, and so time lapse images of the subsurface can be obtained. There is experimental evidence that subsurface biological activity can significantly change velocities in a wet subsurface environment^33,34^, so efficient time-lapse PI surveys might be able to detect bio-activity near the surface.

The PI technique can be used as a real-time monitoring tool to characterize the fluid flow in different materials. It has the potential for using seismic experiments to rapidly detect the time-varying properties of structures, such as engineering materials, buildings, mines, dams and the time-varying subsurface properties of planetary soils (see Fig. 7). The properties of soils can be tested in the field by depositing fluids onto the soils and monitoring the changing patterns in the velocity tomograms. This method can also be used an efficient means for conducting efficient seismic surveys of subsurface mineral content in planetary bodies such as the Moon or Mars.

The time-lapse PI technique is the first seismic method that can estimate the velocity variations in a large object at time-lapse intervals of less than a few minutes and provides at least an order-of-magnitude increase in temporal resolution of a time-lapse seismic experiment. As with many fields of science, an order-of-magnitude increase in the resolution capabilities of an imaging device can lead to significant advances in that field. We believe that PI will lead to such advances in geophysical and engineering imaging of subsurface fluid flow.

## Supplementary Information

Supplementary Information

## Data Availability

The datasets generated during and/or analyzed during the current study are available from the corresponding author on reasonable request.
