# Supplementary material for: Near-surface real-time seismic imaging using parsimonious interferometry
Source: Sci Rep. 2021 Mar 30;11:7194. doi: 10.1038/s41598-021-86531-5 (PMC8010075; doi:10.1038/s41598-021-86531-5)
Supplement: Supplementary file 1 — Supplementary Information [file 41598_2021_86531_MOESM1_ESM.docx]

Near-surface Real-time Seismic Imaging using Parsimonious Interferometry

Sherif M. Hanafy^1^, Hussein Hoteit^2^, Jing Li^3,*^, and Gerard T. Schuster^2^

^1^ King Fahd University of Petroleum and Minerals, CPG, Department of Geosciences, Dhahran 34464, Saudi Arabia.

^2^ King Abdullah University of Science and Technology, Division of Physical Science and Engineering, Thuwal 23955-6900. Saudi Arabia.

^3^ College of Geo-exploration Science and Technology, Jilin University, Changchun, China

***^*^*** *sherif.geo@gmail.com*

**Supplemental Material**

**Recorded Seismic Data**

Figure A1 shows a sample of the recorded seismic data. The data are recorded using a sledgehammer hitting a small metallic plate. The sample shot gather shown in Figure A1 is recorded at offset 18 m.


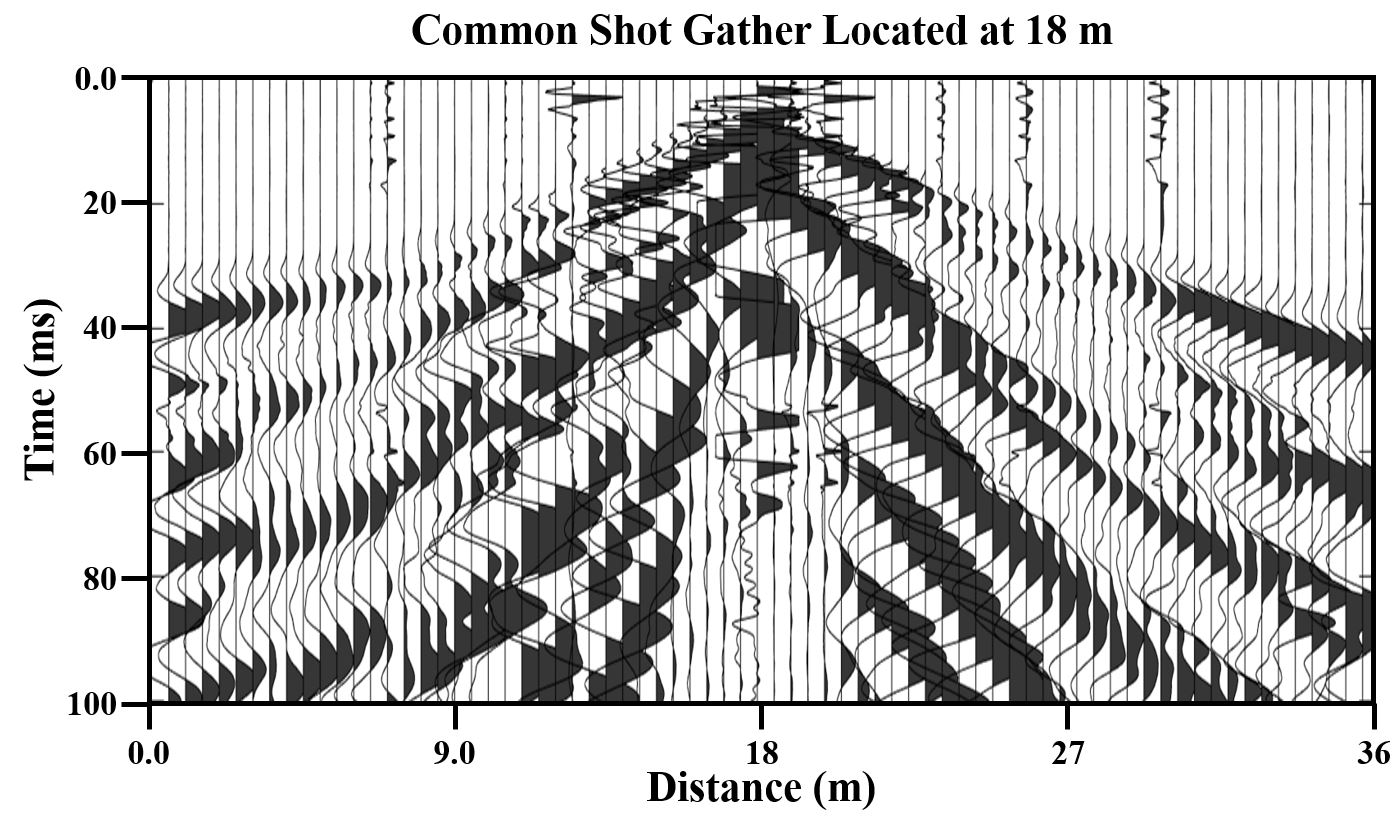


**Fig. A1.** Sample of the recorded seismic data. The shot is located at offset 18 m. The data shows a high signal-to-noise ratio and the first arrival times are clear to pick. MatLab 2018b (<https://www.mathworks.com/>) is used to plot this Figure.

**Parsimonious surface-wave interferometry method**

The virtual trace at C generated by a virtual source at B in Figure A2 can be created by correlating the trace recorded at C with the trace recorded at B if they are both excited by the source at A. The dispersion curves of the resulting virtual surface waves can be inverted to reconstruct the S-velocity model.


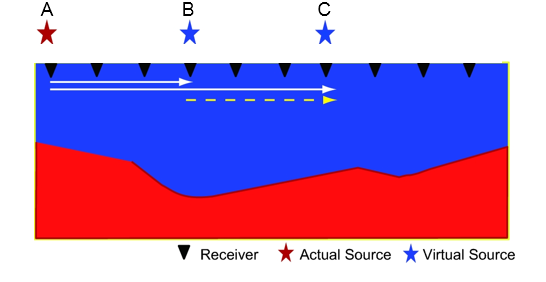
**Fig. A2.** The recordings at **B** and **C** can be correlated with one another to give the virtual trace Φ (**B, C**)**_A_** at **C** as if the source was located at **B.** Here we assume that the fundamental-mode Rayleigh waves have been isolated in the recordings, and the yellow dashed line indicates the ray diagram for this virtual Rayleigh wave.

Similar to virtual refraction arrivals generated by the PI method, virtual shot gathers containing surface waves can also be generated at every geophone in the recording survey^11^. The dispersion curves if the virtual surface waves can be inverted to reconstruct the S-velocity model. As an example, if a source is at **A** in Figure A2, then the traces associated with a pair of geophones at **B** and **C** can be correlated with one another to produce the virtual trace Φ(**B**,**C**)_A_ recorded at **C** as if it were generated by a source at **B (**see Figure A3). In this case the virtual arrivals of interest are the fundamental-mode Rayleigh waves because we assume that they have been isolated in the records. Their signal-to-noise ratio can be enhanced by generating a new virtual trace Φ(**B**, **C**)_A’_ for a new source at **A’**, which is to the left of **B** in Figure A2. This new trace can be added to the virtual trace Φ (**B**, **C**)_A_ to enhance the signal-to-noise ratio to give the stacked trace Φ (**B**, **C**)_A_ + Φ (**B**, **C**)_A’._ Repeating this procedure for different sources gives N virtual shot gathers, with a virtual source at each of the N geophones. The dispersion curves from the virtual shot gathers can be computed and the fundamental modes can be inverted to give the S-velocity model. Additional details and numerical simulations of the surface-wave parsimonious interferometry method are given in Li et al.^11^ .

**CSGs and Dispersion Curves from Sand Dune Data**

A typical shot gather recorded on the sand dune is shown in Figure A3a, and its associated dispersion curve is displayed in Figure A3b. The usable frequency ranges for the fundamental mode denoted by the white dashed line is between 25 Hz and 90 Hz.


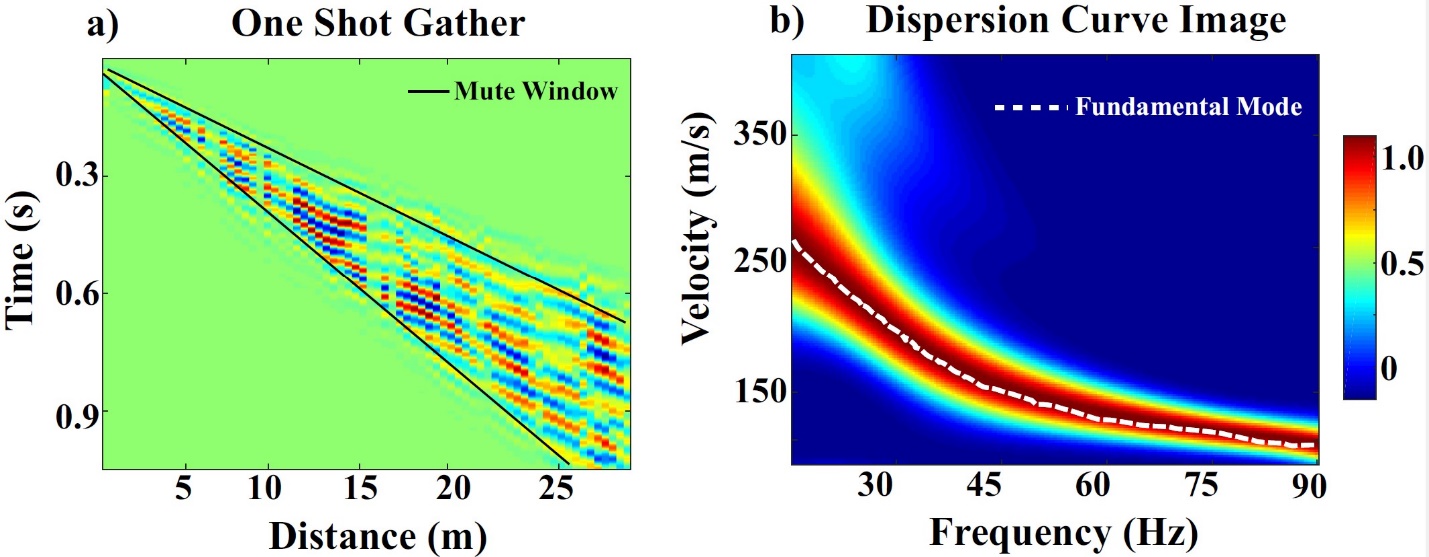


**Fig. A3.** a) Common shot gather and b) associated dispersion curve obtained by applying a temporal FFT and Radon transform^11^ to the windowed data in a). MatLab 2018b (<https://www.mathworks.com/>) is used to plot this Figure.

**Background Velocity Tomograms**

The first arrival times in the 72 shot gathers are picked and inverted by refraction traveltime tomography^10^ to give the background P-velocity tomogram in Figure A4a. The Rayleigh waves are windowed and the associated fundamental dispersion curves (e.g. Figure A3b) are picked and inverted using wave equation dispersion inversion^11^ to give the background S-velocity tomogram shown in Figure A4b.


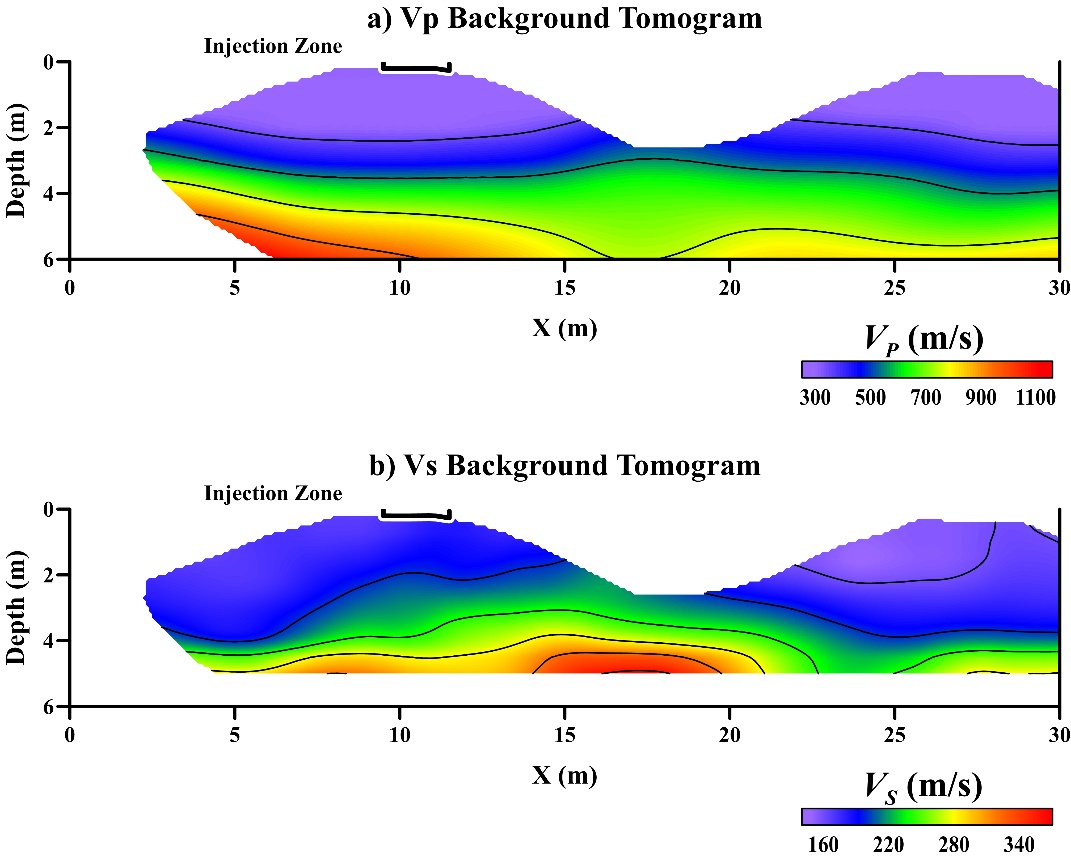


**Fig. A4.** Background a) P-velocity and b) S-velocity tomograms inverted from the 72 CSGs recorded over the sand dune prior to water injection. Surfer 11 (https://www.goldensoftware.com/products/surfer) is used to plot this Figure.

**Comparison of Virtual and Actual Refraction Traveltimes**

**Figure 1b.** For traces that contain only the fundamental mode of the Rayleigh wave, the recordings at **B** and **C** can be correlated with one another to give the virtual trace F(**B,C**)**_A_** at **C**, as if the source was located at **B**. The yellow dashed line indicates the ray diagram of this virtual Rayleigh wave.

The virtual refraction traveltimes computed by PI are compared to refraction traveltimes recorded from the background data set. Figure A5a displays the first-arrival traveltimes of the recorded 72 shot gathers as a function of the shot and receiver indices; when the shot index *i* is equal to the receiver index *i* this means that the *i^th^* source is at the *i^th^* geophone location so that the first arrival traveltime is at t=0. The picked 72x72=5184 traveltimes are compared to the same number of virtual traveltimes, which are generated from the traveltimes of only 6 shot gathers (located at receivers 1, 15, 29, 43, 57, and 72) displayed in Figure 5b. These times are inserted into equation 3 (in the main text) to generate 5184 virtual traveltimes (see Figure A5c). Figure A5d shows the histogram of the differences between the background (Figure A5a) and virtual (Figure A5c) traveltimes, where they agree with one another by about ±3 ms. This indicates a good match between the background and virtual traveltimes, where the dominant period of the source wavelet is 24 ms. The error of ±3 ms is within the picking accuracy of the recorded data.


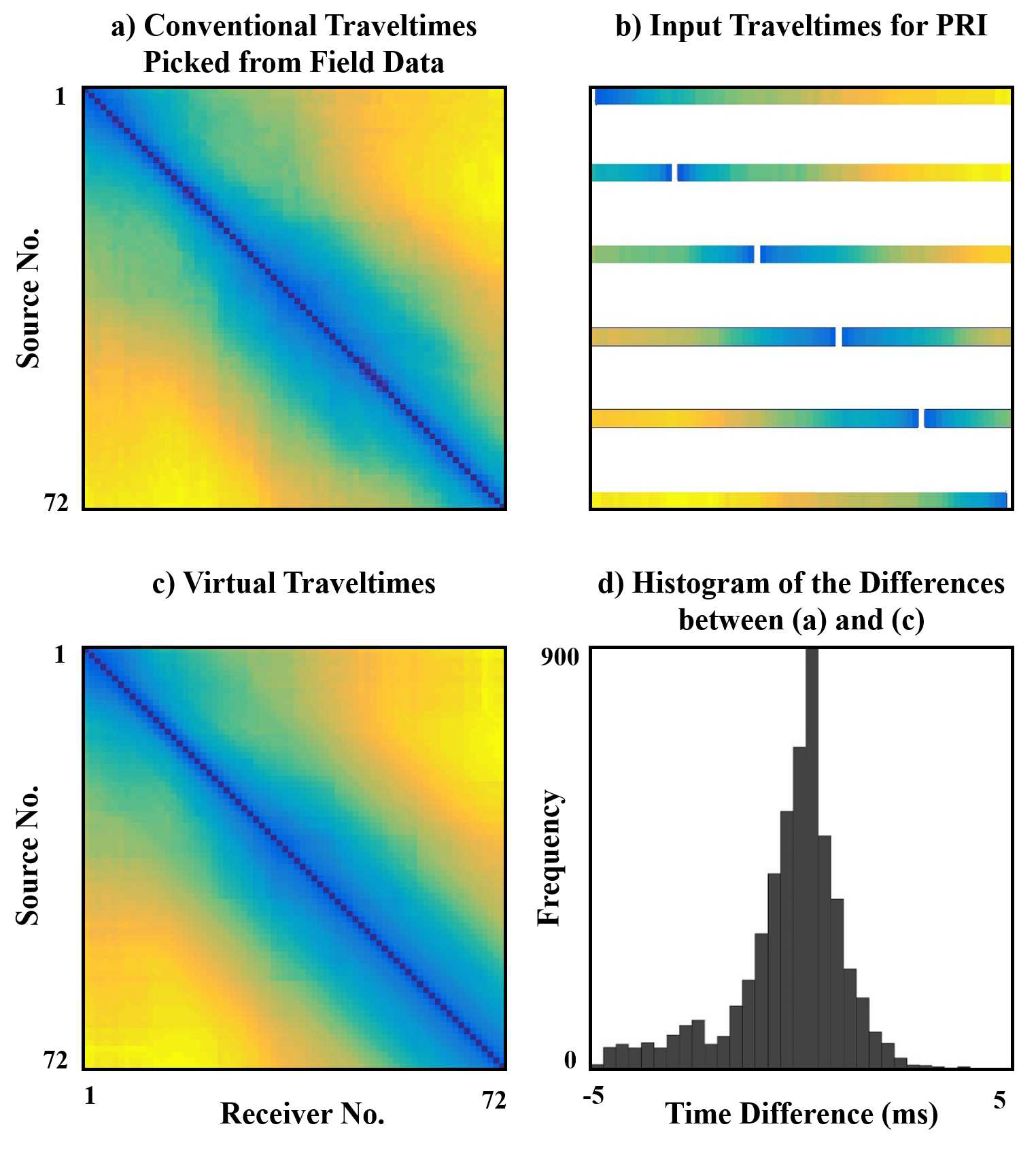
**Fig. A5.** Validation of the refraction PI method using the refraction traveltimes picked from the background seismic data set. a) The traveltimes of the 72-recorded shot gathers, b) the 6-shot gathers used as input to the Parsimonious Refraction Interferometry (PRI) method, c) the virtual first-arrival traveltimes calculated by equation 3 in the main text, and d) the histogram showing the differences between the a) background and c) the virtual first-arrival traveltimes, where the absolute time differences are no more than about 3 ms. MatLab 2018b (<https://www.mathworks.com/>) is used to plot this Figure.

**Resolution Limits of P-velocity and S-velocity Tomograms**

**Figure 1b.** For traces that contain only the fundamental mode of the Rayleigh wave, the recordings at **B** and **C** can be correlated with one another to give the virtual trace F(**B,C**)**_A_** at **C**, as if the source was located at **B**. The yellow dashed line indicates the ray diagram of this virtual Rayleigh wave.

The S-velocity and P-velocity tomograms differ in their spatial resolution limits Δx, which is estimated to be Δx_S_ $\propto$ 0.5 V_s_ /f_s_ =0.5x200 m/s ÷25 Hz = 4.0 m for the S-tomogram at the depth of about 4 meters and Δx_p_ $\propto$ 0.5 V_p_ /f_p_ = 0.5x300 m/s ÷90 Hz = 1.7 m for the P-velocity tomogram. Here, f^P^ = 90 Hz is the highest recorded frequency associated with the refracted arrivals and fs = 25 Hz is the lowest frequency in the recorded Rayleigh waves that penetrate to the deepest depth of about 4 meters. The theoretical resolution limit of the P-velocity tomogram is almost half that of the S-velocity tomogram, so we use the P-velocity tomogram to regularize the S-velocity inversion of the dispersion data. This means that we devise a penalty function for the objective function of dispersion inversion so that the shape of the resulting S-velocity tomogram will be roughly consistent with that of the P-velocity tomogram. The regularization constraint we selected is that the direction of the velocity gradient of the S-velocity tomogram should agree with that of the P-velocity tomogram^13^. This reflects the fact that both tomograms should be sensitive to an increase in water saturation of the sand, but their sensitivities do not have to be the same magnitude. This type of gradient constraint has proven to be very effective in combining medical images obtained by different types of scanners^13^.

Checkerboard tests similar to those in [11] are used to assess the resolution limits of our tomography methods for a given recording geometry and source wavelet. Figures A6a-A6b depict the P-velocity and S-velocity checkerboard tomograms obtained from synthetic data generated by finite-difference solutions to the wave equation. The background velocity model is that for a smooth version of models in Figure A2, where the checkerboard velocity perturbations displayed in Figures A6a and Figures A6b are superimposed on the background velocity models. The checkerboards are of dimension 3x3 m^2^ and differ by +/- 5% velocity from the background velocity models in Figure A2. The refraction traveltimes and dispersion curves are picked and inverted to give, respectively, the P-velocity and S-velocity tomograms in Figures A6c and A6d. These tomograms suggest that the tomography methods can achieve a theoretical spatial resolution of at least 3 meters for the sand dune data. The tomograms suggest an acceptable accuracy in velocity to a depth of about 4 meters.


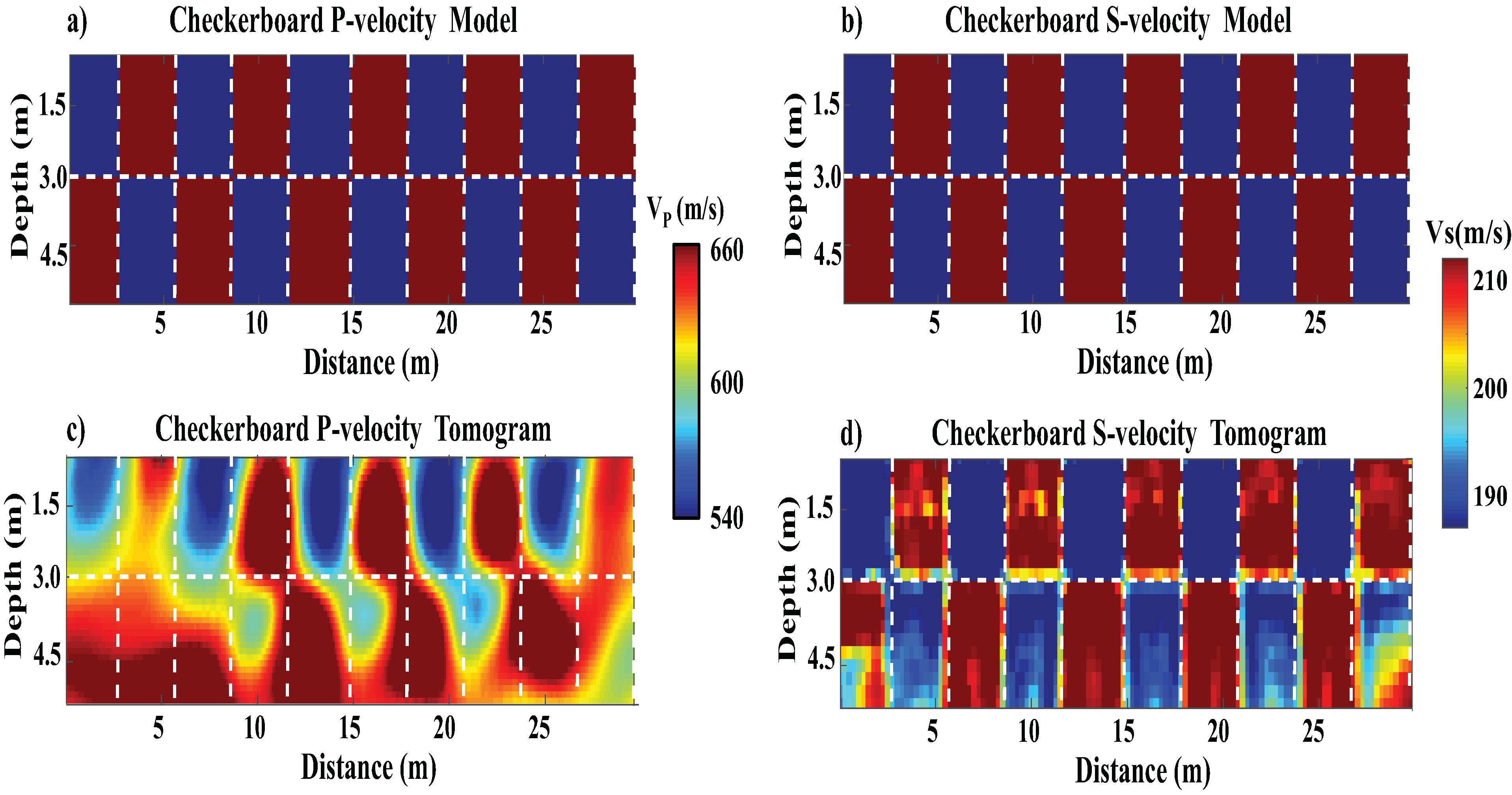


**Fig. A6.** Checkerboard test, a) P-velocity and b) S-velocity initial models. Checkerboard tomograms in c) and d) obtained by inverting, respectively, the refraction traveltimes and dispersion curves computed from the synthetic data. These data were synthetically recorded with the same source-receiver parameters from the sand-dune experiment. Six shot gathers (each with 72 receivers) are the input, the PI method generates 66=72-6 virtual shot gathers, and they are inverted by the refraction traveltime tomography and wave-equation dispersion (WD) methods. The source wavelet here is a Ricker wavelet peaked at 50 Hz. MatLab 2018b (<https://www.mathworks.com/>) is used to plot this Figure.

After smoothing the tomogram, the effective cell width in the slowness model is $\boldsymbol{\Delta}\boldsymbol{x}$ = 0.5 m and the estimated picking error for the P-wave traveltimes is $\boldsymbol{\delta\tau}$ = 4 ms. Therefore the maximum slowness error due to picking errors after one iteration of a gradient descent method is $\boldsymbol{\Delta}\boldsymbol{e}_{\boldsymbol{s}}=\frac{\boldsymbol{\delta\tau}}{\boldsymbol{\Delta}\boldsymbol{x}}=\frac{\mathbf{0}.\mathbf{004}}{\mathbf{0}.\mathbf{5}}=\mathbf{0}.\mathbf{008}$ s/m. Since the minimum P-slowness value in the tomogram is $\frac{\mathbf{1}}{\mathbf{950}}=\mathbf{1}.\mathbf{053x1}\mathbf{0}^{-\mathbf{3}}$ s/m, this constitutes a maximum slowness error of 12.6 % for the P-slowness tomogram. Similar considerations show that the percent error for the S-slowness tomogram is around 16.4 %. Slowness errors due to incomplete source and receiver coverage can be estimated by synthetic tests and the computation of the model covariance matrix.

**Reconstructions of Synthetic Sand Dune Model**

**Figure 1b.** For traces that contain only the fundamental mode of the Rayleigh wave, the recordings at **B** and **C** can be correlated with one another to give the virtual trace F(**B,C**)**_A_** at **C**, as if the source was located at **B**. The yellow dashed line indicates the ray diagram of this virtual Rayleigh wave.

To test the efficacy of our reconstruction methods, we use finite-difference solutions of the wave equation to generate the point-source responses of the elastic velocity models described in Figures A7a-A7b. We use the same source-receiver geometry employed for the sand-dune experiment, and invert both the refraction traveltimes and dispersion curves. The resulting tomograms are shown in Figure A7c-A7d, and show acceptable accuracy when compared to the true models.

To enhance the consistency of the S-velocity and P-velocity tomograms, we use the velocity gradient constraint as a penalty function for inverting the S-velocity tomogram. That is, the direction of the S-velocity gradient ∇Vs should be parallel to the P-velocity gradient ∇Vp so that the penalty function λ||∇Vs x ∇Vs|| is minimized^11^; here, λ is the scalar damping factor that balances the conflicting desires for a small data misfit and consistency between the P- and S-velocity tomograms. The results for this constrained inversion are shown in Figure A7e and A7f. In both tomograms, the constrained velocity tomograms show a better resemblance to the true velocity models than the unconstrained ones in A7c-A7d. Figure A8 is similar to Figure A7, except the subsurface velocity model has changed because the fluid has flowed to different portions of the model. Both Figures A7 and A8 demonstrate that the constrained velocity tomograms can provide more accurate velocity changes reconstructed from time-lapse data.

**Fig. A7.** Model test 1: P- and S-velocity water-injection models in a) and b), and the associated tomograms in c) and d). e) and f) are the tomograms using the cross-gradient as a regularization term. The input data were generated by finite-difference solutions to the elastic wave equation, and the recording geometry is the same as that for the sand-dune experiment with 72 receivers and 72 shots on the surface. MatLab 2018b (<https://www.mathworks.com/>) is used to plot this Figure.

**Fig. A8.** Same as Figure A7, except the water has moved to different portions of the subsurface. MatLab 2018b (<https://www.mathworks.com/>) is used to plot this Figure.

**Topography Test for Surface-wave Dispersion in the Sand Dune experiment**

**Figure 1b.** For traces that contain only the fundamental mode of the Rayleigh wave, the recordings at **B** and **C** can be correlated with one another to give the virtual trace F(**B,C**)**_A_** at **C**, as if the source was located at **B**. The yellow dashed line indicates the ray diagram of this virtual Rayleigh wave.

In this section we tested the effect of the topography on the dispersion curves. Figure A9 shows the synthetic P- and S-wave velocity models with topography. The model size is similar to the actual model of the sand dune experiment, where the maximum topographic change is 1.5m. We used the finite-difference solutions to the elastic wave equation to generate 72 synthetic shot gathers with geometry similar to our field experiment. Figure A10a shows a shot gather assuming no topography change while Figure A10b shows a shot gather with topography change as shown in Figure A9. Figures A11a and A11b show the dispersion curves corresponding to data without (Figure A10a) and with (Figure A10b) topographic changes, respectively. We can observe minor changes in the dispersion curves, however, it is minimum and does not affect the velocity inversion results.

**Fig. A9.** P- and S-velocity inject water models with topography in a) and b). The model size is same with the sand dune experiment. The maximum topographic change is 1.5m. MatLab 2018b (<https://www.mathworks.com/>) is used to plot this Figure.

**Fig. A10.** a) and b) are the shot gather without and with topography changes, respectively. They were generated by finite-difference solutions to the elastic wave equation, and the recording geometry is the same as that for the sand-dune experiment with 72 receivers and 72 shots on the surface.

**Fig. A11.** Comparison of the dispersion curve images without and with topographic changes. MatLab 2018b (<https://www.mathworks.com/>) is used to plot this Figure.
